# Bcr-Abl: one kinase, two isoforms, two diseases

**DOI:** 10.18632/oncotarget.20874

**Published:** 2017-09-14

**Authors:** Sina Reckel, Oliver Hantschel

**Affiliations:** Oliver Hantschel: Swiss Institute for Experimental Cancer Research, School of Life Sciences, École Polytechnique Fédérale de Lausanne, Lausanne, Switzerland

**Keywords:** leukemia, targeted therapies, tyrosine kinase inhibitor, functional proteomics, imatinib/gleevec

Over the past decades, hundreds of oncogenic driver mutations that encode aberrantly activated proteins, were identified in various tumors. A predominant class of oncoproteins is constitutively activated protein kinases that cause uncontrolled cell proliferation and necessitated the development of small-molecule chemical inhibitors that block kinase activity. Among these, imatinib (Gleevec) represents the showcase example of molecular targeted therapies as it potently and quite selectively inhibits the tyrosine kinase Bcr-Abl that drives different human leukemias. The presence of Bcr-Abl is the result of a chromosomal translocation event forming the Philadelphia chromosome that fuses the breakpoint cluster region (BCR) gene to the N-terminus of the Abelson tyrosine kinase (ABL1) gene [1]. Depending on the translocation breakpoint in the BCR gene, different Bcr-Abl protein isoforms exist with the most common members Bcr-Abl p210 and p190. The p210 isoform is 501 amino acids and thus 25% longer than p190 and it is the hallmark of chronic myeloid leukemia (CML) [2]. In CML, Bcr-Abl is the sole oncogenic driver and treatment with imatinib and successors leads to durable remissions in most patients. Additionally, Bcr-Abl is expressed in 20-30% of adult B-cell acute lymphoblastic leukemia (B-ALL), where about one quarter of cases express the p210- and three quarters express the p190-isoform of Bcr-Abl. In contrast to CML, B-ALL is often accompanied by additional mutagenic events and survival is dramatically low due to relapse and kinase inhibitor resistance [3]. The molecular basis for the different disease association of p210 and p190 is not understood, as previous research mainly focused on the p210 isoform and no direct comprehensive comparison of p210 and p190 was available. In a parallel study, we and a second research group have now shed light on the differential p210 and p190 signaling networks using a combination of quantitative interaction- and phosphoproteomics revealing unexpectedly large differences [4], [5]. In our comprehensive study, we identified 56 proteins that commonly interact with both Bcr-Abl isoforms, 34 proteins preferentially interacted with p190 and 13 proteins with p210. Among the most striking differences, we found all subunits of the AP2 adaptor complex that regulates clathrin-mediated endocytosis, which preferentially interact with p190. On the other hand, the non-canonical tyrosine phosphatase Sts1 was enriched with p210. Mapping of the tyrosine-phosphoproteome revealed a common Bcr-Abl signature of more than 500 phosphotyrosine (pY) sites, along with ∼100 pY sites that were differentially regulated between the two Bcr-Abl isoforms. In particular, stronger activation of the Stat5 transcription factor and the Erk1/2 kinases was observed with p210, and the Src family kinase Lyn was activated by p190, which indicated activation of distinct signaling pathways by p210 and p190 Bcr-Abl. Many differential hits in our study constitute functionally critical nodes for oncogenic transformation, leukemogenesis and kinase inhibitor resistance. In particular, Stat5 is absolutely required for CML initiation and disease maintenance and Stat5 upregulation mediates kinase inhibitor resistance [6]. In contrast, Lyn was shown to be required for B-ALL formation in mouse models and Lyn upregulation also mediated imatinib resistance [7].

A possible molecular explanation for these surprising findings could be a different level of kinase activation between Bcr-Abl p210 and p190. Although it is important to note, that p210 and p190 are identical in all domains of Abl, including the enzymatic kinase domain. Accordingly, we found no difference in degree and sites of p210 and p190 autophosphorylation or *in vitro* kinase activity of Bcr-Abl. As an alternative scenario, Bcr-Abl p210 could have a different subcellular localization than p190 owing to its different domain composition. Bcr-Abl p210 would thereby encounter another subset of the proteome than p190, resulting in the strong differences in interacting proteins that we observed. Likewise, the kinase domain of p210 and p190 would be able to phosphorylate a different subset of substrates. Future studies will need to clarify the precise structural differences of p210 and p190 and possible differential localization.

Our findings nicely demonstrate the power and sensitivity of modern functional proteomics approaches to validate previously known and identify novel interaction partners and signaling pathways downstream of important oncoproteins. Despite the close similarity of the two Bcr-Abl isoforms, we mapped differential activation of tyrosine kinase and phosphatase pathways that may drive CML or B-ALL. Targeting those pathways will provide a more coherent understanding of the mechanisms of Bcr-Abl signaling and leukemic transformation, as well as responses to kinase inhibitors. Importantly, the observed differential signaling networks of p210 and p190 suggest new approaches to improve the prognosis of patients with kinase inhibitor resistance and offer more effective treatment options, in particular for B-ALL patients. Our data has revealed differential activation of prominent, druggable cell signaling pathways including the Jak-Stat and Ras-Raf-Mek-Erk pathways, as well as the Src family kinases, which could be inhibited using Jak2, Raf/Mek or Src inhibitors that are in clinical use. In particular, a combination with Bcr-Abl tyrosine kinase inhibitors could be of benefit, albeit the best, possible synergistic combination remains to be identified.
